# Supplementary material for: Molnupiravir inhibits Bourbon virus infection and disease-associated pathology in mice
Source: J Virol. 2025 Sep 5;99(10):e00740-25. doi: 10.1128/jvi.00740-25 (PMC12548437; doi:10.1128/jvi.00740-25)
Supplement: Supplemental figures — Figures S1 to S5. [file jvi.00740-25-s0001.docx]

**SUPPLEMENTARY DATA**

**Supplementary Figure 1. The viability of the A549 cells was minimally affected by NHC**. A549 cells were incubated with different concentrations of NHC. Cell viability at 48 h was measured by using the Cell Titer Blue assay. DMSO was used as the mock control, and Triton X-100 was used as the positive control for cell death. Values are means (± standard error of the mean) of the cell viability from three experiments performed in duplicate.

**Supplementary Figure 2. Favipiravir inhibits BRBV infection in cell culture*.*** A549 cells inoculated with BRBV (MOI of 0.01) in the presence or absence of different concentrations of favipiravir. The virus titer in the culture supernatant was quantified at 12, 24, 48, and 72 h. Values are means (± standard error of the mean) of the virus titer from three experiments performed in duplicate. **, *P <* 0.01, *** *P <* 0.005 by one-way ANOVA on the area under the curves fit by linear regression of log-transformed virus titer over time and compared with Dunnett’s multiple comparisons. The dotted line represents the limit of detection at 200 FFU/mL.

**Supplementary Figure 3: Molnupiravir is well tolerated by *Ifnar1* ^-/-^ mice.** Uninfected *Ifnar1*^-/-^ mice (n = 2) were treated orally with 150 mg/kg (mid dose) of molnupiravir twice daily for 8 days. Weight change was monitored for 8 days. Values are the means (± standard error of the mean) of the treated group.

**
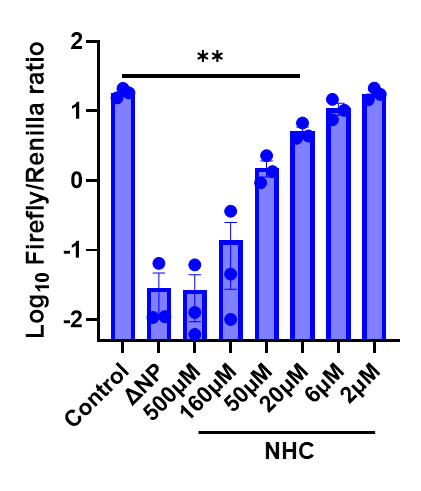
**

**Supplementary Figure 4: Favipiravir inhibits BRBV polymerase activity.** The effect of different concentrations of favipiravir on the BRBV polymerase activity was quantified using the BRBV mini-genome assay. Expression plasmids encoding for the BRBV polymerases (PB2, PB1, PA) and NP of BRBV and Renilla luciferase, plus the reporter construct (Firefly luciferase flanked by the 3' and 5' UTR of segment 2 of BRBV) were transfected into 293T cells in the presence or absence of different concentrations of favipiravir. At 48 h post-transfection, the ratio of firefly luciferase to renilla luciferase activities was measured. Values are geomeans (± standard error of the geomean) of the luciferase activity from three experiments performed in duplicate. **, *P <* 0.01, by one-way ANOVA and Dunnett’s multiple comparisons.

**
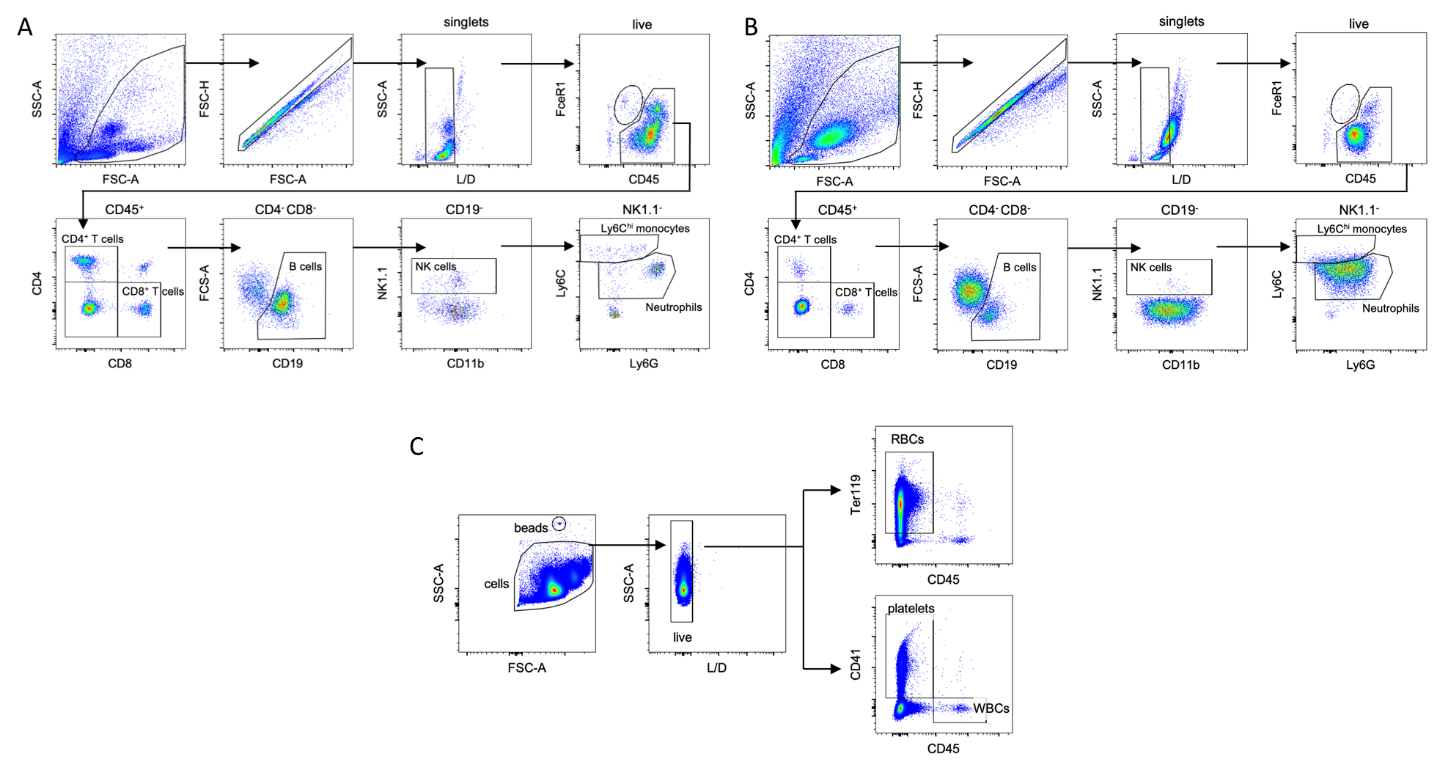
**

**Supplementary Figure 5:** (**A-C**) Flow cytometry gating strategy for (**A**) uninfected spleen cells (**B**) infected spleen cells and (**C**) peripheral blood RBC, WBC, and platelets.
